# Effect of “edible clay” (takere) suspension on serum lipid profiles and atherogenic indices of normal Wistar rats

**DOI:** 10.1002/fsn3.910

**Published:** 2019-02-06

**Authors:** Catherine C. Ikewuchi, Jude C. Ikewuchi, Ugochukwu K. Ezeka, Mercy O. Ifeanacho

**Affiliations:** ^1^ Faculty of Science Department of Biochemistry University of Port Harcourt Port Harcourt Nigeria

**Keywords:** atherogenic index of plasma, atherogenic indices, edible clay, lipid profiles, takere

## Abstract

This study was undertaken to ascertain the effect of an aqueous suspension of a commonly available preparation of edible clay (“takere”) on serum lipid profiles and atherogenic indices of normal Wistar rats. Ninety‐five adult Wistar rats of average weight of 100 g were assigned into seven groups. Group 1 (Baseline) was immediately sacrificed at the commencement of study; Group 2 (Control) daily received distilled water, orally; and Groups 3 to 7 received via the same route (per body weight), 125 mg/kg (T125), 250 mg/kg (T250), 500 mg/kg (T500), 1,000 mg/kg (T1000), and 2,000 mg/kg (T2000) of the takere suspension, respectively, for 28 days. In week 1, the treatments significantly (*p* < 0.05) lowered the levels of serum triglyceride (by T250, T1000, and T2000), VLDL cholesterol (by T250, T1000, and T2000), and atherogenic index of plasma (AIP; by T250) and significantly (*p* < 0.05) raised the levels of serum HDL (T250), LDL (T250 and T2000), non‐HDL (T2000) cholesterols, atherogenic coefficient (AC; T2000), cardiac risk ratio (CRR; T2000), and Castelli's risk index II (CRI‐II; T2000) of the rats. In week 2, the treatments significantly (*p* < 0.05) lowered the levels of serum triglyceride (T2000), HDL (T125, T500, T1000, and T2000), VLDL (T2000) cholesterols and significantly (*p* < 0.05) raised levels of serum LDL (T125, T1000, and T2000), non‐HDL (T125, T1000, and T2000) cholesterols, AC (T125, T500, and T1000), CRR (T125, T500, and T1000), CRI‐II (T125 and T1000), AIP (T125, T500, and T1000) of the rats. In week 4, the treatments significantly (*p* < 0.05) raised the levels of serum total (T500 and T2000), HDL (T2000), non‐HDL (T500 and T1000) cholesterols, AC (T500), CRR (T500), and CRI‐II (T500). This result indicates that the consumption of *takere* suspension may have adverse effects on serum lipid profiles and atherogenic indices of Wistar rats, at least at the doses administered in this study.

## 1 INTRODUCTION

The deliberate consumption of clay (geophagy or geophagia) is a common habit of humans, most often in rural or pre‐industrial societies (Abrahams, Davies, Solomon, Trow, & Wragg, 2013). According to the Encyclopedia of Medical Anthropology, “although geophagy is known among the general population, it is quite common among pregnant women” (Lex & Abler, 2004). It is more established and prevalent in Asia and Africa (Hooda, Henry, Seyoum, Amstrong, & Fowler, 2002; Norman, Binka, & Godi, 2015; Wilson, 2003), although practiced in many continents of the world (Abrahams & Parsons, 1997; Aufreiter, Hancock, Mahaney, Stambolic‐Robb, & Sanmugadas, 1997; Grigsby, Thyer, Waller, & Johnston, 1999; Höllriegl et al., 2007; Woode & Hackman‐Duncan, 2014; Woywodt & Kiss, 2002). Poverty, famine (Woywodt & Kiss, 2002), spiritual, religious (Ferrell, 2008), ritual (Hunter, 1993), physiological (Vermeer & Ferrell, 1985), psychological, nutritional, medical (Dominy, Davoust, & Minekus, 2004), cultural (Norman et al., 2015), and social needs (Geissler et al., 1999) are among the prominent factors responsible for the continued sustenance of this practice.

While some studies have attributed no harmful effect to the consumption of edible clay (Obialo et al., 2001; Vermeer & Frate, 1979), some have elicited the benefits attributable to clay consumption practice (Dominy et al., 2004; Reichardt et al., 2011), and others still frown at its detrimental and aberrant effects (Ekanem, Ekong, Eluwa, Igiri, & Osim, 2015; Ekong, Peter, Ekanem, & Osim, 2015a; Woode & Hackman‐Duncan, 2014). The opponents of edible clay consumption posit that, in addition to its content of mineral elements and heavy metals, it may also contain a variety of pathogenic organisms such as bacteria (Aghamirian & Ghiasian, 2009; Bisi‐Johnson, Obi, & Ekosse, 2010; Okereke & Okereke, 2016) and parasitic geohelminths (Abrahams, 2002; Ogomaka, 2015).

Earlier, Reichardt et al. (2011) reported that edible clay consumption was beneficial for individuals undergoing extreme nutritional conditions. Their study showed that kaolin supplementation lowered percentage body fat contents in rats. However, despite this and the plethora of reports on geophagia or clay‐eating, nothing has been reported on its effect on serum lipid profiles and atherogenic indices, which are potent predictors of cardiovascular disease risk. Therefore, this study investigated the effect of the consumption of edible clay on the serum lipid profiles and atherogenic indices of normal Wistar rats.

## 2 MATERIALS AND METHODS

### 2.1 Collection of edible clay and preparation of edible clay suspension

Blocks of edible clay (*takere*) were purchased from a local market in Port Harcourt, Nigeria. They were ground into powder using a manually operated grinder. A 40 g sample of the powder was dissolved in 441 ml of distilled water, and the mixture was stirred continuously to produce a well‐dispersed suspension. The suspension was stored and was stirred prior to every administration.

### 2.2 Experimental design

Wistar rats of both male and female sex weighing between (90 and 120 g) were obtained from the Animal Breeding Unit of the College of Veterinary Medicine, University of Nigeria, Nsukka, and kept in well‐ventilated plastic/stainless steel cages. They were left under laboratory conditions to acclimatize for 2 weeks and were allowed feed (commercial feed) and water ad libitum. They were then sorted into seven groups (Table 1) such that the difference in mean weights of the groups was ≤1.5 g. Group 1 (Baseline) consisted of five rats; Groups 2 to 7 were each further subdivided into three subgroups of five rats tagged week 1, week 2, and week 4. After an additional 1‐week acclimatization, the Group 1 (Baseline) was immediately sacrificed, before commencing the administration of the suspension, which lasted for 4 weeks. Group 2 (Control) orally received distilled water, daily, while Groups 3–7 received various doses of the suspension ranging, respectively, from 125, 250, 500, 1,000, and 2,000 mg/kg (Table 1), through the same route. The choice of dosage ranges was informed by the consideration of the earlier use of 200 mg/kg by Ekanem et al. (2015), and the report by Ekong et al. (2015a), Ekong, Peter, Ekanem, and Osim (2015b) showed that no mortalities were recorded at a dose of 5,000 mg/kg. The animals were weighed weekly. Five rats were sacrificed from each of Groups 2–7, on the first, second, and fourth week of the 28‐day experimental period, and blood samples were collected by cardiac puncture, into anticoagulant‐free bottles. The serum were collected and stored for use in the assay.

**Table 1:** Treatment schedule of the experimental groups

S/N	Identity	Treatment
1	Group 1 (Baseline)	a
2	Group 2 (Control)	Distilled water (p.o.)
3	Group 3 (T125)	125 mg/kg suspension (p.o.)
4	Group 4 (T250)	250 mg/kg suspension (p.o.)
5	Group 5 (T500)	500 mg/kg) suspension (p.o.)
6	Group 6 (T1000)	1,000 mg/kg) suspension (p.o.)
7	Group 7 (T2000)	2,000 mg/kg suspension (p.o.)

*Notes*

p.o.: “per os” or orally.

a The baseline group was sacrificed before treatment.

### 2.3 Measurement of serum lipid profiles

Serum triglyceride, total cholesterol, and HDL cholesterol were assayed using Randox test kits (Randox Laboratories Ltd., UK). The glycerol released from triglycerides by lipases was oxidized with the resultant release of hydrogen peroxide, whose breakdown by peroxidase, in the presence of 4‐chlorophenol, converted 4‐aminophenazone to quinoneimine (the indicator) whose concentration was determined spectrophotometrically at 500 nm. The free cholesterol and those released by enzymatic hydrolysis was oxidized with concomitant release of hydrogen peroxide, whose breakdown converted 4‐aminoantipyridine to quinoneimine (the indicator) whose concentration was determined spectrophotometrically at 500 nm. A mixture of phosphotungstic acid and magnesium ions was used to quantitatively precipitate and remove LDL, VLDL, and chylomicron fractions, after which the cholesterol content of the HDL fraction, remaining in the supernatant, was determined, as for total cholesterol.

### 2.4 Estimation of serum VLDL, LDL, and non‐HDL cholesterol concentrations

Serum VLDL and LDL cholesterol (LDLC and VLDLC) concentrations were calculated using the Friedewald formula (Friedewald, Levy, & Friedrickson, 1972) as follows:

[LDL cholesterol] (mmol/L) = [Total cholesterol] − [HDL cholesterol] − [triglyceride]/2.2
[VLDL cholesterol] (mmol/L) = [Triglyceride]/2.2

The non‐HDL cholesterol concentrations were determined according to a formula earlier reported by Ikewuchi, Ikewuchi, and Ifeanacho (2014) as follows.

[Non‐HDL cholesterol] = [Total cholesterol] − [HDL cholesterol]

### 2.5 Determination of atherogenic indices

The atherogenic indices were calculated using the following formulae:

Cardiac risk ratio = [Total cholesterol]/[HDL cholesterol] (Ikewuchi & Ikewuchi, 2009)
Atherogenic coefficiecnt = ([Non‐HDL cholesterol])/([HDL cholesterol]) (Ikewuchi, Ikewuchi, Ifeanacho, Igboh, & Ijeh, 2013)
Castelli's risk index II = [LDL cholesterol]/[HDL cholesterol] (Bhardwaj, Bhattacharjee, Bhatnagar, & Tyagi, 2013)
Atherogenic index of plasma = log ([Triglyceride])/([HDL cholesterol]) (Ikewuchi, Onyeike, Uwakwe, & Ikewuchi, 2011)

### 2.6 Statistical analysis

Descriptive statistics were carried out on the data generated. Results were expressed as the mean ± standard error of the mean. They were analyzed using one‐way analysis of variance, and significant difference of the means was determined using least significant difference test. Student's *t* test was used to compare the values to their corresponding baseline values. Probability values less than 0.05 (*p *<* *0.05) were considered significant. Graphs were drawn using Microsoft Office Excel, 2010 software.

## 3 RESULTS

### 3.1 Effect on serum lipid profiles

**Figure 1:**
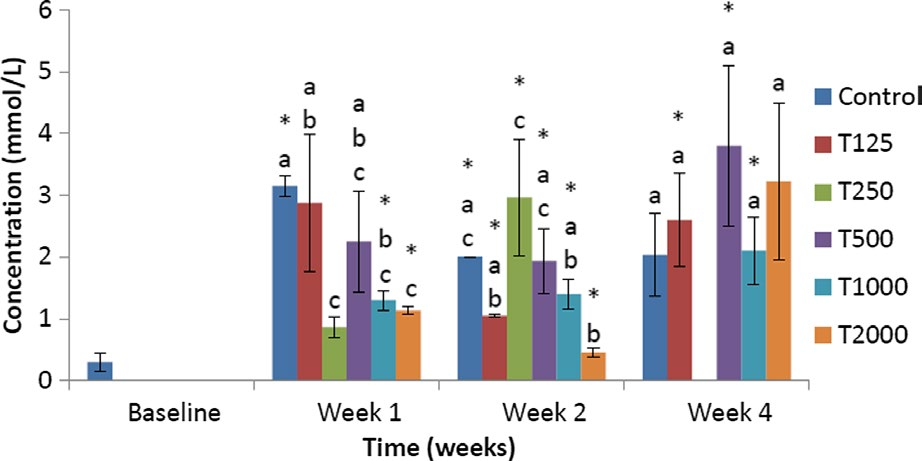
Effect of *takere* suspension on serum triglyceride concentrations of normal Wistar rats. Values are expressed as mean ± standard error of the mean, *n* = 5. Values in the same week with different superscripts (a, b, c), differ significantly at *p *<* *0.05. *p <* *0.05 compared to baseline value

The effect of *takere* suspension on serum triglyceride concentrations of normal Wistar rats is presented in Figure 1. In week 1, the serum triglyceride concentration of the control was significantly (*p *<* *0.05) higher than that of T250, T1000, and T2000, but not significantly higher than that of T125 and T500. Compared to baseline, the serum triglyceride levels of control, T1000, and T2000 were significantly (*p *<* *0.05) higher. In week 2, the serum triglyceride level of the control was significantly (*p *<* *0.05) higher than that of T2000, but not significantly higher than those of T125, T500, and T1000, and not significantly lower than that of T250. Compared to baseline, the serum triglyceride levels of control, T125, T250, T500, T1000, and T2000 were significantly (*p *<* *0.05) higher. In week 4, the serum triglyceride level of the control was not significantly lower than that of the *takere*‐treated groups. Compared to baseline, the serum triglyceride levels of T125, T500, and T1000 were significantly (*p *<* *0.05) higher.

**Figure 2:**
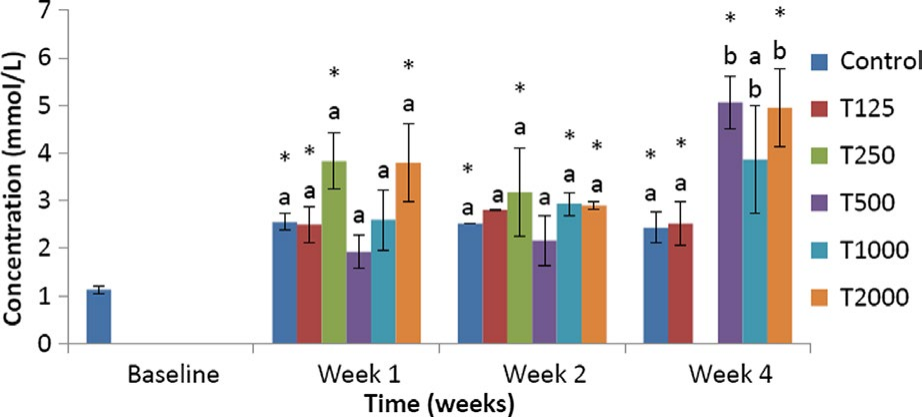
Effect of *takere* suspension on serum total cholesterol concentrations of normal Wistar rats. Values are expressed as mean ± standard error of the mean, *n* = 5. Values in the same week with different superscripts (a, b), differ significantly at *p *<* *0.05. *p <* *0.05 compared to baseline value

As shown in Figure 2, in week 1, the serum total cholesterol concentration of the control was not significantly lower than that of T250, T1000, and T2000 and not significantly higher than that of T125 and T500. Compared to baseline, the serum total cholesterol concentrations of control, T125, T250, and T2000 were significantly (*p *<* *0.05) higher. In week 2, the serum total cholesterol level of control was not significantly higher than that of T500 and not significantly lower than that of T125, T250, T1000, and T2000. Compared to baseline, the serum total cholesterol levels of control, T250, T1000, and T2000 were significantly (*p *<* *0.05) higher. In week 4, the serum total cholesterol concentration of the control was significantly (*p *<* *0.05) lower than that of T500 and T2000, but not significantly lower than that of T125 and T1000. Compared to baseline, the serum total cholesterol levels of control, T125, T500, and T2000 were significantly (*p *<* *0.05) higher.

**Figure 3:**
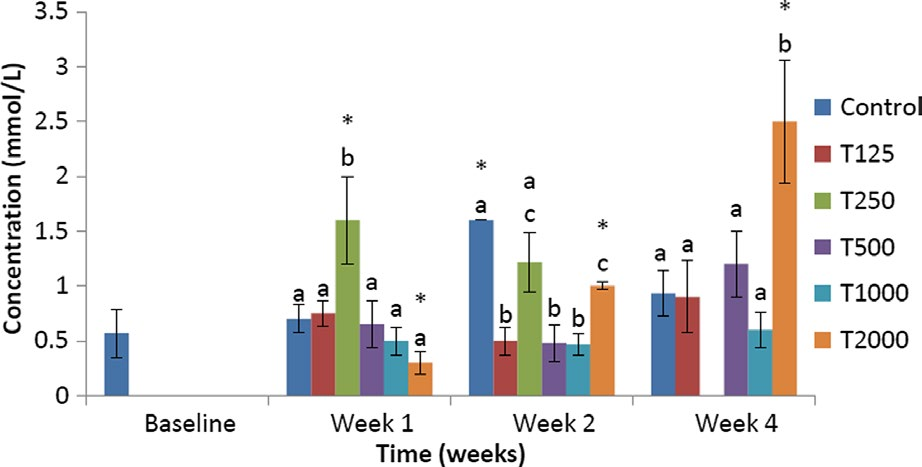
Effect of *takere* suspension on serum HDL cholesterol concentrations of normal Wistar rats. Values are expressed as mean ± standard error of the mean, *n* = 5. Values in the same week with different superscripts (a, b, c) differ significantly at *p *<* *0.05. *p <* *0.05 compared to baseline value

The effect of *takere* suspension on serum HDL cholesterol concentrations of normal Wistar rats is shown in Figure 3. In week 1, the serum HDL cholesterol level of the control was significantly (*p *<* *0.05) lower than that of T250 and not significantly lower than that of T125, but was not significantly higher than that of T500, T1000, and T2000. Compared to baseline, that of T250 was significantly (*p *<* *0.05) higher, while that of T2000 was significantly (*p *<* *0.05) lower. In week 2, the serum HDL cholesterol level of the control was significantly (*p *<* *0.05) higher than that of T125, T500, T1000, and T2000, but not significantly higher than that of T250. Compared to baseline, those of the control and T2000 were significantly (*p *<* *0.05) higher. In week 4, the serum HDL cholesterol level of the control was significantly (*p *<* *0.05) lower than that of T2000, and not significantly lower than that of T500, but was not significantly higher than that of T125 and T1000. Compared to baseline, that of T2000 was significantly (*p *<* *0.05) higher.

**Figure 4:**
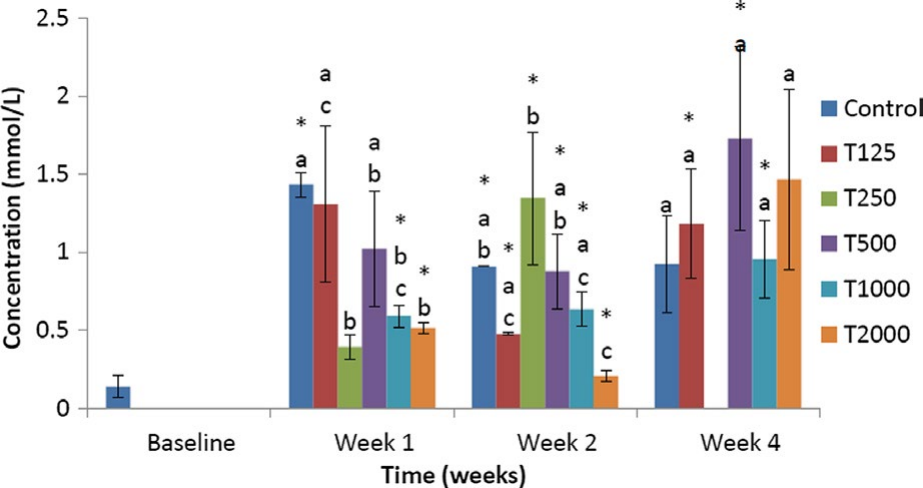
Effect of *takere* suspension on serum VLDL cholesterol concentrations of normal Wistar rats. Values are expressed as mean ± standard error of the mean, *n* = 5. Values in the same week with different superscripts (a, b, c) differ significantly at *p *<* *0.05. *p <* *0.05 compared to baseline value

The effect of *takere* suspension on serum VLDL cholesterol concentrations of normal Wistar rats is presented in Figure 4. In week 1, the serum VLDL cholesterol level of the control was significantly (*p *<* *0.05) higher than that of T250, T1000, and T2000, and not significantly higher than that of T125 and T500. Compared to baseline, the serum VLDL cholesterol levels of control, T1000, and T2000 were significantly (*p *<* *0.05) higher. In week 2, the serum VLDL cholesterol level of the control was significantly (*p *<* *0.05) higher than that of T2000 and not significantly higher than that of T125, T500, and T1000, but was not significantly lower than that of T250. Compared to baseline, the serum VLDL cholesterol levels of the control, T125, T250, T500, T1000, and T2000 were significantly (*p *<* *0.05) higher. In week 4, the serum VLDL cholesterol level of the control was not significantly lower than that of T125, T250, T500, T1000, and T2000. Compared to baseline, the serum VLDL cholesterol levels of T125, T500, and T1000 were significantly (*p *<* *0.05) higher.

**Figure 5:**
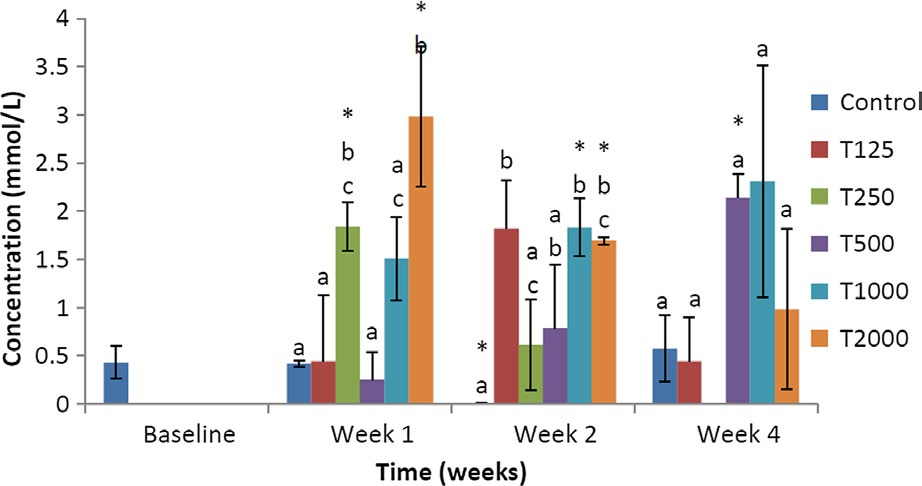
Effect of *takere* suspension on serum LDL cholesterol concentrations of normal Wistar rats. Values are expressed as mean ± standard error of the mean, *n* = 5. Values in the same week with different superscripts (a, b, c), differ significantly at *p *<* *0.05. *p <* *0.05 compared to baseline value

The effect of *takere* suspension on serum LDL cholesterol concentrations of normal Wistar rats is presented in Figure 5. In week 1, the serum LDL cholesterol level of the control was significantly (*p *<* *0.05) lower than that of T250 and T2000 and not significantly lower than that of T125 and T1000; but was not significantly higher than that of T500. Compared to baseline, the serum LDL cholesterol levels of T250 and T2000 were significantly (*p *<* *0.05) higher. In week 2, the serum LDL cholesterol level of the control was significantly (*p *<* *0.05) lower than that of T125, T1000, and T2000 and not significantly lower than that of T250 and T500. Compared to baseline, the serum LDL cholesterol levels of T1000 and T2000 were significantly (*p *<* *0.05) higher, while those of control were significantly (*p *<* *0.05) lower. In week 4, the serum LDL cholesterol level of the control was not significantly lower than that of T500, T1000, and T2000, but was non‐significantly higher than that of T125. Compared to baseline, the serum LDL cholesterol level of T500 was significantly (*p *<* *0.05) higher.

**Figure 6:**
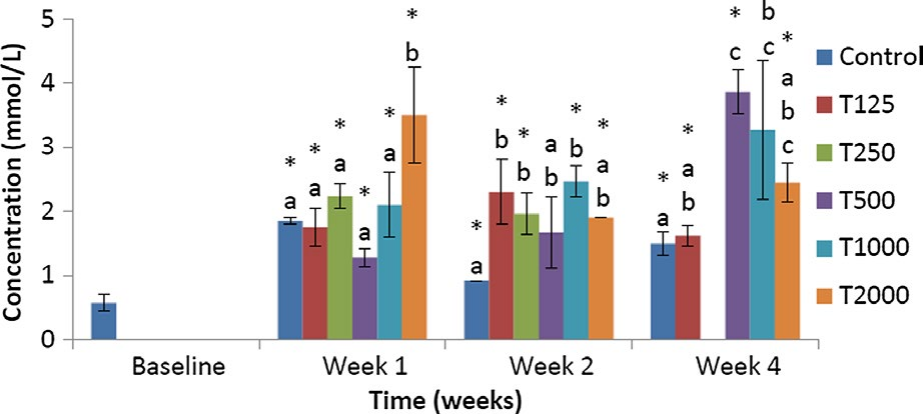
Effect of *takere* suspension on serum non‐HDL cholesterol concentrations of normal Wistar rats. Values are expressed as mean ± standard error of the mean, *n* = 5. Values in the same week with different superscripts (a, b, c) differ significantly at *p *<* *0.05. *p <* *0.05 compared to baseline value

The impact of *takere* suspension on serum non‐HDL cholesterol concentrations of normal Wistar rats is presented in Figure 6. In week 1, the serum non‐HDL cholesterol level of the control was significantly (*p *<* *0.05) lower than that of T2000 and not significantly lower than that of T250 and T1000, but was not significantly higher than that of T125 and T500. Compared to baseline, the serum non‐HDL cholesterol levels of the control, T125, T250, T500, T1000, and T2000 were significantly (*p *<* *0.05) higher. In week 2, the serum non‐HDL cholesterol level of the control was significantly (*p *<* *0.05) lower than that of T125, T250, and T1000 and not significantly lower than that of T500 and T2000. Compared to baseline, the serum non‐HDL cholesterol levels of the control, T125, T250, T1000, and T2000 were significantly (*p *<* *0.05) higher. In week 4, the serum non‐HDL cholesterol level of the control was significantly (*p *<* *0.05) lower than that of T500 and T1000 and not significantly lower than that of T125 and T2000, but was not significantly higher than that of T125 and T1000. Compared to baseline, the serum non‐HDL cholesterol levels of the control, T125, T500, and T2000 were significantly (*p *<* *0.05) higher.

### 3.2 Effects on atherogenic indices

**Figure 7:**
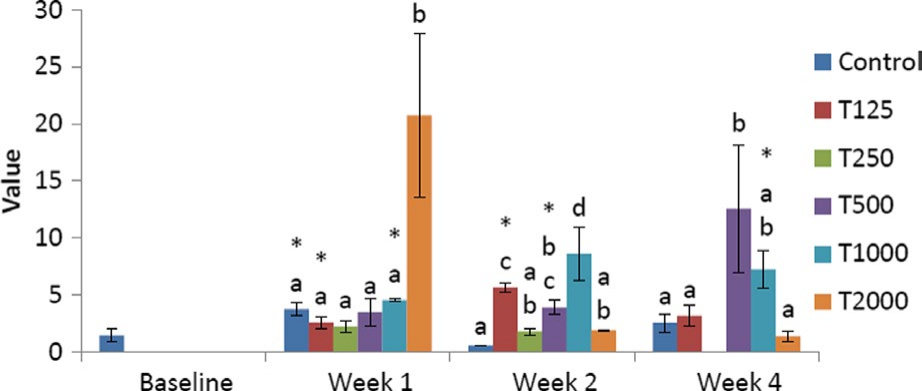
Effect of *takere* suspension on the atherogenic coefficients of normal Wistar rats. Values are expressed as mean ± standard error of the mean, *n* = 5. Values in the same week with different superscripts (a, b, c) differ significantly at *p *<* *0.05. *p <* *0.05 compared to baseline value

The influence of *takere* suspension on atherogenic coefficients of normal Wistar rats is presented in Figure 7. In week 1, the atherogenic coefficient of the control was significantly (*p *<* *0.05) lower than that of T2000 and not significantly lower than that of T1000 and T2000, but was non‐significantly higher than that of T125, T250, and T500. Compared to baseline, those of the control, T125, and T1000 were significantly (*p *<* *0.05) higher. In week 2, the atherogenic coefficient of the control was significantly (*p *<* *0.05) lower than that of T125, T500, and T1000 and not significantly lower than that of T250 and T2000. Compared to baseline, those of T125 and T500 were significantly (*p *<* *0.05) higher. In week 4, the atherogenic coefficient of the control was significantly (*p *<* *0.05) lower than that of T500 and not significantly lower than that of T125 and T1000, but was non‐significantly higher than that of T2000. Compared to baseline, that of T1000 was significantly (*p *<* *0.05) higher.

**Figure 8:**
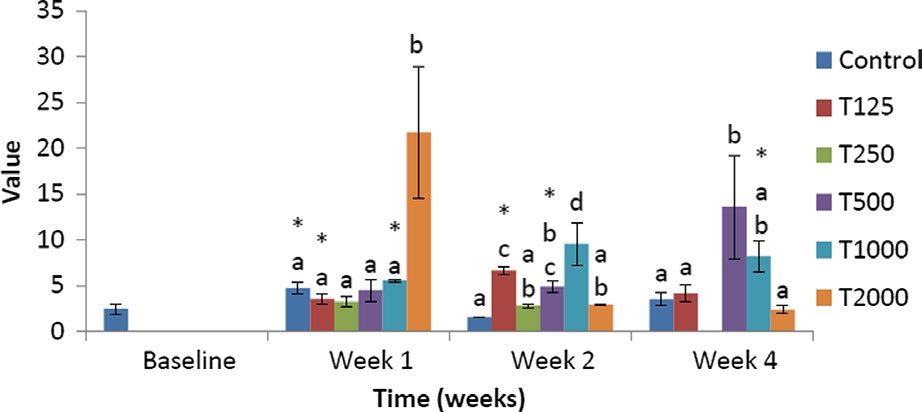
Effect of *takere* suspension on the cardiac risk ratios of normal Wistar rats. Values are expressed as mean ± standard error of the mean, *n* = 5. Values in the same week with different superscripts (a, b, c) differ significantly at *p *<* *0.05. *p <* *0.05 compared to baseline value

The effect of *takere* suspension on cardiac risk ratios of normal Wistar rats is presented in Figure 8. In week 1, the cardiac risk ratio of the control was significantly (*p *<* *0.05) lower than that of T2000 and not significantly lower than that of T1000, but was not significantly higher than that of T125, T250, and T500. Compared to baseline, those of the control, T125, and T1000 were significantly (*p *<* *0.05) higher. In week 2, the cardiac risk ratio of the control was significantly (*p *<* *0.05) lower than that of T125, T500, and T1000 and not significantly lower than that of T250 and T2000. Compared to baseline, those of T125 and T500 were significantly (*p *<* *0.05) higher. In week 4, the cardiac risk ratio of the control was significantly (*p *<* *0.05) lower than that of T500 and not significantly lower than that of T125 and T1000, but was not significantly higher than that of T2000. Compared to baseline, that of T1000 was significantly (*p *<* *0.05) higher.

**Figure 9:**
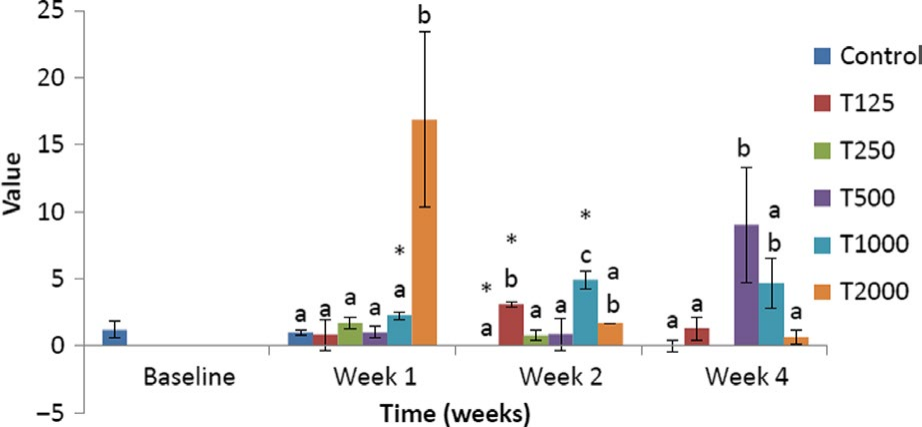
Effect of *takere* suspension on the Castelli's risk index II of normal Wistar rats. Values are expressed as mean ± standard error of the mean, *n* = 5. Values in the same week with different superscripts (a, b, c) differ significantly at *p *<* *0.05. *p <* *0.05 compared to baseline value

The impact of *takere* suspension on Castelli's risk index II of normal Wistar rats is presented in Figure 9. In week 1, the Castelli's risk index II of the control was significantly (*p *<* *0.05) lower than that of T2000 and not significantly lower than that of T250, T500, and T1000, but was not significantly higher than that of T125. Compared to baseline, Castelli's risk index II of T1000 was significantly (*p *<* *0.05) higher. In week 2, Castelli's risk index II of the control was significantly (*p *<* *0.05) lower than that of T125 and T1000 and not significantly lower than that of T250, T500, and T2000. Compared to baseline, Castelli's risk index II of T125 and T1000 was significantly (*p *<* *0.05) higher, while that of control was significantly (*p *<* *0.05) lower. In week 4, Castelli's risk index II of the control was significantly (*p *<* *0.05) lower than that of T500 and not significantly lower than that of T125, T1000, and T2000.

**Figure 10:**
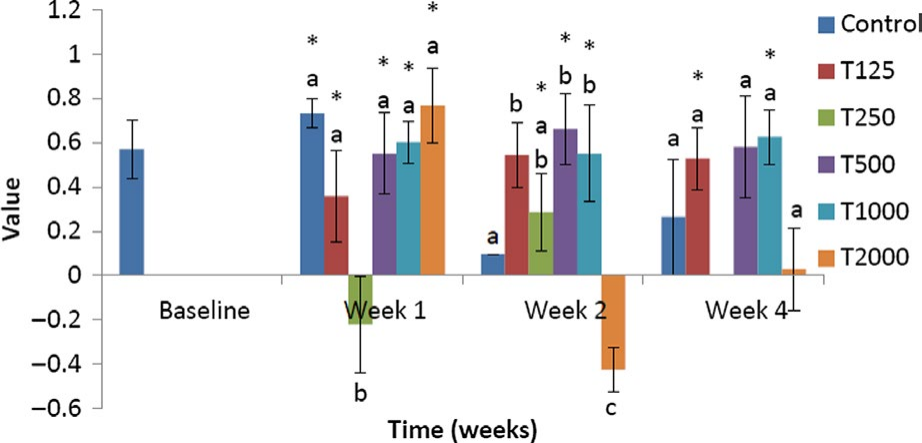
Effect of *takere* suspension on the atherogenic index of plasma of normal Wistar rats. Values are expressed as mean ± standard error of the mean, *n* = 5. Values in the same week with different superscripts (a, b, c) differ significantly at *p *<* *0.05. *p <* *0.05 compared to baseline value

The effect of *takere* suspension on atherogenic index of plasma of normal Wistar rats is presented in Figure 10. In week 1, the atherogenic index of plasma of the control was significantly (*p *<* *0.05) higher than that of T250 and not significantly higher than that of T125, T500, and T1000, but was not significantly lower than that of T2000. Compared to baseline, those of the control, T1000, and T2000 were significantly (*p *<* *0.05) higher, while those of T125 and T500 were significantly (*p *<* *0.05) lower. In week 2, the atherogenic index of plasma of the control was significantly (*p *<* *0.05) lower than those of T125, T500, and T1000 and not significantly lower than that of T250, but is significantly (*p *<* *0.05) higher than that of T2000. Compared to baseline, that of T500 was significantly (*p *<* *0.05) higher, while those of T250 and T1000 were significantly (*p *<* *0.05) lower. In week 4, the atherogenic index of plasma of the control was not significantly lower than that of T125, T500, and T1000, but was not significantly higher than that of T2000. Compared to baseline, that of T1000 was significantly (*p *<* *0.05) higher, while that of T125 was significantly (*p *<* *0.05) lower.

## 4 DISCUSSION

### 4.1 Moderation of serum lipid profiles

In weeks 1 and 2, the treatments lowered the serum triglyceride levels of the rats. This may be an indication of the hypolipidemic or cardioprotective activity of the suspension, because reductions in serum or plasma levels of triglyceride have been considered to reduce risk of developing atherosclerosis and other cardiovascular diseases (McBride, 2007, 2008). This lipid‐lowering effect is in conformity with the earlier report by Reichardt et al. (2011), of the lowering of percentage body fat composition in rats by pica.

In week 4, the treatments (T500 and T2000) raised the serum total cholesterol levels of the rats. This may be ominous, as raised serum or plasma total cholesterol is a risk factor for cardiovascular diseases (Lichtenstein et al., 2006). The treatments, in week 1 (T250) and week 4 (T2000), raised the serum HDL cholesterol levels of the rats. This elevated serum HDL cholesterol portends potential for the reduction of cardiovascular risk (Assmann & Gotto, 2004; Rang, Dale, Ritter, & Moore, 2005), in the treated animals.

The treatments, in week 1 (T250, T1000, and T2000) and week 2 (T2000), lowered the serum VLDL cholesterol levels of the rats. This may be an indication of the hypolipidemic or cardioprotective activity of the suspension, because reductions in serum or plasma levels of VLDL cholesterol have been reported to reduce risk of developing atherosclerosis and other cardiovascular diseases (Shen, 2007).

The treatments, in week 1 (T250 and T2000) and week 2 (T125, T1000, and T2000), raised the serum LDL cholesterol levels of the rats. Recall, that reductions in serum or plasma levels of LDL cholesterol have been considered to reduce risk of developing atherosclerosis and other cardiovascular diseases (Shen, 2007). Conversely, raised serum or plasma level of LDL cholesterol is a risk factor for cardiovascular disease (Ademuyiwa, Ugbaja, Idumebor, & Adebawo, 2005; Lichtenstein et al., 2006). Thus, the increase produced by the suspension in this study may portend predisposition to increased risk of cardiovascular diseases.

The treatments, in week 1 (T2000), week 2 (T125, T250, and T1000), and week 4 (T500 and T1000), raised the serum non‐HDL cholesterol levels of the rats. Non‐HDL cholesterol is an indicator of atherogenic apolipoprotein B containing lipoproteins such as low‐density lipoproteins, very low‐density lipoproteins, and intermediate‐density lipoproteins (Hermans, Sacks, Ahn, & Rousseau, 2011). Thus, it is analogous to Apo‐B in assessing atherogenic cholesterol and lipoprotein burden (Hermans et al., 2011). This makes it a better predictor of cardiovascular disease risk than a simple measure of LDL cholesterol (Brunzell et al., 2008; Nimmanapalli, Kasi, Devapatla, & Nuttakki, 2016; Peters, 2008). Therefore, the raised serum levels of non‐HDL cholesterol produced by the administration of the *takere* suspension may signify predisposition of its consumers to higher risks of cardiovascular disorders.

### 4.2 Moderation of atherogenic indices

The study by Nimmanapalli et al. (2016) showed that there is a significant correlation and association between atherogenic indices and nitric oxide, a marker of endothelial dysfunction. The findings of Bhardwaj et al. (2013) suggested that atherogenic coefficient reflects atherogenic potential of the entire spectrum of lipoprotein fractions and hence predicts cardiovascular risk. According to Dobiášová, Frohlich, Šedová, Cheung, and Brown (2011) and Dobiášová and Frohlich (2001), atherogenic index of plasma reflects the actual composition of the lipoprotein spectrum and correlates positively with the fractional esterification rate of HDL and inversely with low‐density lipoprotein particle size. They further stated that it is a good predictor of cardiovascular risk. Thus, people with high atherogenic index of plasma have a higher risk for coronary artery disease (Wan et al., 2015). In this study, the treatment raised the various atherogenic indices of the treated rats. Therefore, the raised atherogenic indices produced by the *takere* suspension, portend a likely predisposition to cardiovascular diseases.

## 5 CONCLUSION

These results show that the consumption of edible clay (*takere*) suspension altered the lipid profiles and atherogenic indices of Wistar rats. Though some of these moderations were positive, most of them were negative and connote adverse effects. Thus, this implies that the consumption of edible clay may have an overall adverse effect on the serum lipid profiles and atherogenic indices.

## CONFLICT OF INTEREST

The authors declare that they have no conflicts of interest concerning this article.

## ETHICAL REVIEW

This study was approved by University of Port Harcourt Research Ethics Committee, as part of the MSc research project for Ezeka, Ugochukwu K. All experimental procedures were performed in accordance with the National Institute of Health Guide for the Care and Use of Laboratory Animals (NIH Publications No. 80‐23, revised in 1996).
